# Diagnostic performance of a vessel-length-based method to compute the instantaneous wave-free ratio in coronary arteries

**DOI:** 10.1038/s41598-020-57424-w

**Published:** 2020-01-24

**Authors:** Kyung Eun Lee, Gook Tae Kim, Eui Cheol Jung, Eun Seok Shin, Eun Bo Shim

**Affiliations:** 1 Department of Mechanical and Biomedical Engineering, Kangwon National University, Chuncheon, Kangwon-do 200-701 Republic of Korea; 2 Bio-Convergence Technology Group, Korea Institute of Industrial Technology, Jeju, 63243 Republic of Korea; 3 Division of Cardiology, Department of Internal Medicine, Ulsan University Hospital, University of Ulsan College of Medicine, Dong-gu, Ulsan 682-714 Republic of Korea

**Keywords:** Cardiac device therapy, Interventional cardiology

## Abstract

The instantaneous wave-free ratio (iFR) is a recently introduced vasodilator-free index to assess the functional severity of coronary stenosis in the resting state, while fractional flow reserve (FFR) is the gold standard index in hyperemia. The computed instantaneous wave-free ratio (CT-iFR) is a noninvasive method to estimate iFR using computer simulations. Here, we developed a vessel-length-based CT-iFR method in patient-specific models of coronary arteries. This method was implemented by coupling a three-dimensional computational fluid dynamics model with a lumped parameter model (LPM) of coronary circulation in a non-hyperemic resting state. A time-varying resistance in the LPM was used for the iFR simulation. In total, 50 coronary vessels of 32 patients were computed, and their CT-iFR values were compared with clinically measured iFRs to evaluate the diagnostic performance of the present CT-iFR method. The area under the receiver operating characteristics curve of CT-iFR validation was 0.93. In diagnostic performances of CT-iFR, accuracy, sensitivity, and specificity were 86%, 83.3%, and 86.8%, respectively. These results indicate that this CT-iFR method can be used as a pre-operative aid to establish a percutaneous coronary intervention strategy as a noninvasive alternative to iFR.

## Introduction

Fractional flow reserve (FFR) is widely used to evaluate the functional severity of stenosis in coronary arteries. This index is the ratio of the distal pressure (P_d_) to the proximal pressure (P_a_) through a coronary stenosis under vasodilator (adenosine)-induced hyperemia^1–3^. Here, the pressures are the averaged values over the whole cardiac cycle. On the other hand, the instantaneous wave-free ratio (iFR) is the flow reserve under non-hyperemic resting conditions, thus in the absence of vasodilators^4–6^. In the iFR, the ratio of P_d_/P_a_ is measured during a wave-free period, ranging from mid- to late-diastole, when microvascular resistance is naturally constant and minimized in the cardiac cycle^4–6^. The diagnostic performances of iFR and FFR to determine the need for percutaneous coronary intervention (PCI) have been investigated in many clinical studies^7–11^. These clinical studies have reported that iFR is as accurate as FFR in estimating the hemodynamic severity of coronary stenosis. A recent comparative clinical study of iFR- versus FFR-guided strategies in 2,492 and 2,037 patients revealed that coronary revascularization therapy guided by iFR is comparable to that by FFR, with respect to the risk of major adverse cardiac events^12–14^.

Taylor *et al*.^15^ pioneered non-invasive estimation using computed FFR (CT-FFR; the prefix CT- indicates ‘computed’) in the three-dimensional (3D) geometry of patient-specific coronary arteries reconstructed from computed tomography (CT) images and validated the clinical diagnostic performance and utility of the method in several studies^16–18^. Recently, we also proposed a vessel-length-based CT-FFR method^19–21^ and presented the clinical diagnostic performance of the method^22^. Similar to the CT-FFR approach, computed iFR (CT-iFR) provides the means to noninvasively calculate iFR values in a patient-specific 3D geometry of the coronary arteries. Indeed, the CT-iFR method using commercial software, ANSYS (Canonsburg, USA), was proposed in an existing study^23^; however, the clinical efficacy and diagnostic performance of the method were not fully investigated in the research. To our knowledge, there has been no reported study addressing the clinical validity or performance of the CT-iFR method by comparing CT-iFR values with clinically measured iFRs (M-iFR; the prefix M- indicates ‘pressure-wire measurement’).

In this study, we address the clinical validity and performance of the CT-iFR method. For this purpose, a simulation method to noninvasively estimate iFR values in the 3D geometry of coronary arteries is proposed. This method is implemented by extending the vessel-length-based CT-FFR approach presented in our previous reports^20,21^, in which a computational fluid dynamics (CFD) model of coronary arteries is coupled with a lumped parameter model (LPM) to reflect the effect of coronary microvascular and venous hemodynamics. Here, the parameters of LPM are determined based on the vessel lengths of coronary arteries. To investigate the clinical efficacy and performance of the present CT-iFR method, we simulated the coronary arterial hemodynamics of 32 patients and calculated their iFR values. The simulated CT-iFR values were compared with clinically measured iFR values (M-iFR). Finally, the clinical benefits and limitations of the CT-iFR method are discussed.

## Results

### A clinically measured iFR (M-iFR)

**Figure 1:**
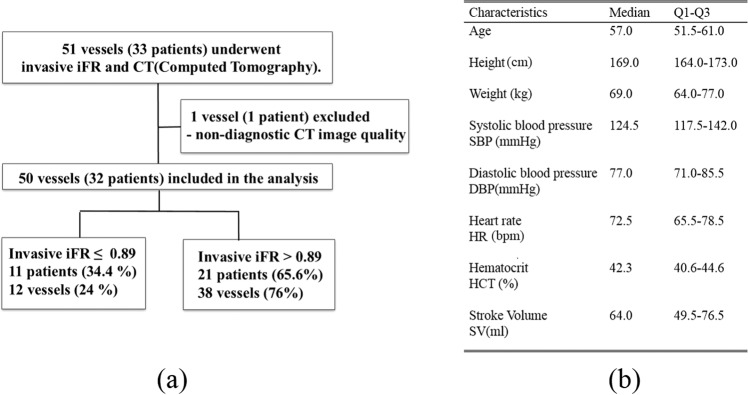
(**a**) Study enrollments for 51 vessels of 33 patients, (1 vessel of 1 patient was excluded). Fifty vessels in 32 patients were included in the study. (**b**) Clinical characteristics of the 32 patients. Clinical data are expressed as medians and interquartile ranges (Q1–Q3). (Unit: bpm, beats per min).

In total, 51 vessels of 33 patients underwent coronary CT angiography and invasive iFR (M-iFR), as presented in Fig. 1(a). One patient was excluded due to the poor diagnostic quality of a blurred CT image; thus, a total of 50 vessels of 32 patients were simulated in this study. Figure 1(a) presents the prevalence of the clinically measured M-iFR values to show the distribution of positive and negative vessel samples. The numbers of vessels of the left anterior descending artery (LAD), left circumflex artery (LCX), and right coronary artery (RCA) in 32 patients were 28 (56%), 15 (30%), and 7 (14%), respectively. Figure 1(b) shows the patients’ physiological characteristics (age, height, weight, blood pressure, heart rate, hematocrit, and stroke volume) with the medians and interquartile ranges (Q1–Q3). Among the 50 vessels included in the study, 38 vessels (76%) were negatively diagnosed (iFR > 0.89), while the number of positively diagnosed vessels (iFR ≤ 0.89) was 12 (24%). Vessels had a stenosis area-based stenosis degree (%) of 72 ± 13 (mean ± standard deviation). Calcifications were observed in 2 vessels (of two patients) among the 50 vessels. The number of positively diagnosed vessels (stenosis degree > 75%) was 22 using CT angiography area-based diagnosis, whereas the number of positively diagnosed vessels (iFR ≤ 0.89) was 12 (24%) using an iFR-based diagnosis.

### Computed iFR (CT-iFR)

**Figure 2:**
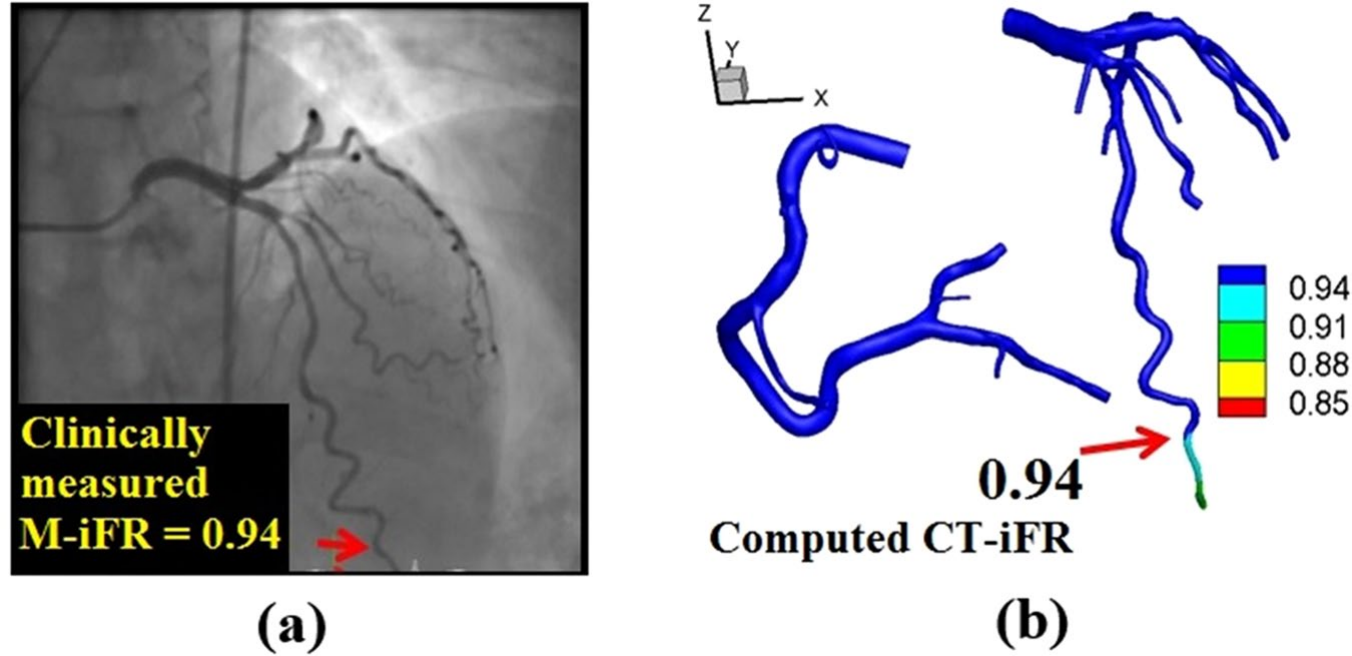
(**a**) Clinically measured instantaneous wave-free ratio (iFR) data (M-iFR) and (**b**) computed iFR results (CT-iFR) in a representative case (Case 1). Here, prefixes M- and CT- refer to ‘clinically measured’ data and ‘computed’ results, respectively.

CT-iFR (the pressure ratio across the stenosis over the wave-free period under non-hyperemic resting conditions) was calculated by a multi-scale simulation technique, as described in our previous papers^20,21^. The method couples the 3D local CFD of patient-specific coronary arterial hemodynamics with the vessel-length-based LPM of coronary microvascular and venous hemodynamics. Figure 2 shows the clinically measured iFR and the CT-iFR for a representative case; Fig. 2(a) shows the M-iFR value at the measured point of iFR marked with a red arrow on the X-ray angiography image, and Fig. 2(b) shows the computed CT-iFR distribution in the patient-specific 3D coronary model. The clinically measured iFR value and the computed iFR showed good agreement. A reduction in the CT-iFR value was observed in the very distal part of the iFR-measured vessel (LAD), due to arterial curvature and arterial tapering close to capillaries (Fig. 2).

### Comparisons between clinically measured iFR (M-iFR) and computed iFR (CT-iFR)

**Figure 3:**
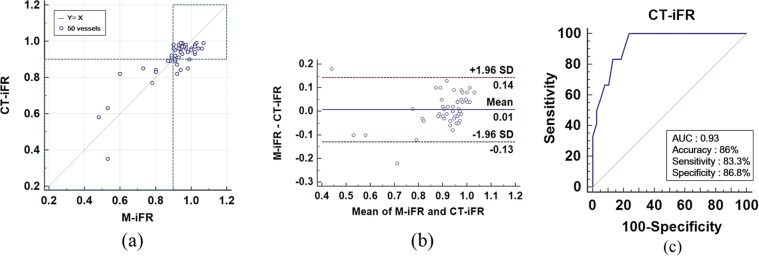
Scatter plot and Bland–Altman plot on a per-vessel basis. (**a**) Scatter plot of iFR between clinical M-iFR data and computed CT-iFR results. (**b**) Bland–Altman plot of iFR between clinical M-iFR data and computed CT-iFR results. (**c**) Receiver-operating characteristic (ROC) curve of CT-iFR using the cut-off M-iFR of ≤ 0.89 on a per-vessel basis.

We compared computed CT-iFR results with clinical M-iFR values on a per-vessel basis (*n* = 50). Figure 3 shows a scatter plot and Bland–Altman plot of the clinical data (M-iFR) and the computed results (CT-iFR) in panels (a) and (b), respectively. Computed CT-iFR results showed good agreement with clinical M-iFR data (*n* = 50) in Fig. 3(a). We also observed good correlation between CT-iFR and M-iFR values (Pearson’s correlation coefficient, *ρ*_*p*_ = 0.85, P < 0.0001). Figure 3(b) presents the Bland–Altman plot, with a mean difference of 0.01 and a 95% limit of agreement over the range −0.13 to 0.14 (*n* = 50). A slight underestimation of CT-iFR relative to M-iFR was evident, as shown in Fig. 3(b).

### Evaluation of diagnosis performances

**Figure 4:**
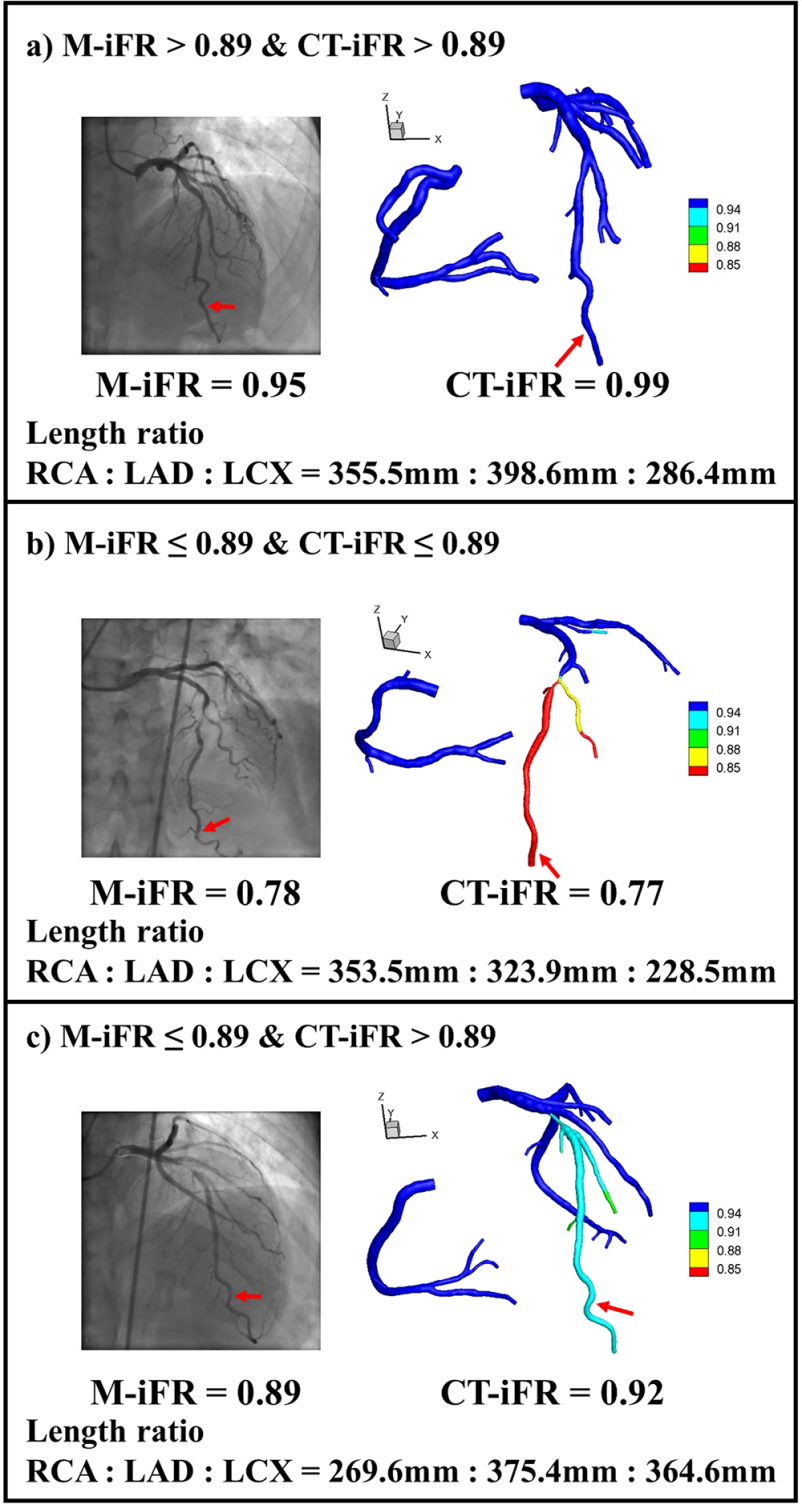
Three representative cases of iFR distribution, including a patient-specific vessel-length ratio, where (**a**) M-iFR > 0.89 and CT-iFR > 0.89, (**b**) M-iFR ≤ 0.89 and CT-iFR ≤ 0.89, and (**c**) M-iFR ≤ 0.89 and CT-iFR > 0.89.

Figure 4 presents the three representative cases of iFR distribution, including the patient-specific vessel-length ratio. In the classification of four groups using the terms of true/false and positive/negative, true or false refers to the assigned classification being correct or incorrect; positive or negative refers to the presence or absence of disease. Figure 4(a) shows a representative case of a true negative group, in which a patient of M-iFR > 0.89 was identified correctly as a patient of CT-iFR > 0.89. Figure 4(b) shows a representative case of a true positive group in which a patient of M-iFR ≤ 0.89 was classified correctly as a patient of CT-iFR ≤ 0.89. There was good diagnostic agreement between M-iFR and CT-iFR readings, as shown in Fig. 4(a,b). Figure 4(c) shows a representative case of a false negative group, in which a patient of M-iFR ≤ 0.89 was identified incorrectly as a patient of CT-iFR > 0.89. Notably, there was diagnostic disagreement between M-iFR and CT-iFR, despite small differences in the values between M-iFR and CT-iFR [Fig. 4(c)]. Figure 4(c) shows the case in which a sick patient was identified as a healthy person. In the case of such intermediate stenosis, small differences can lead to incorrect plans calling for stenting or deference of treatment. A more accurate, non-invasive iFR approach would help to establish better strategies for intermediate stenosis.

The diagnostic performance of CT-iFR was evaluated using receiver operating characteristics (ROC) curve analysis. Figure 3(c) shows the ROC curve of CT-iFR using a cut-off clinical iFR of ≤ 0.89 (*n* = 50). The area under the ROC curve (AUC) value for the diagnostic performance of CT-iFR was 0.933 (95% confidence interval (CI): 0.868–0.998). Diagnostic performances of CT-iFR in terms of the accuracy, sensitivity, and specificity are shown in Fig. 3(c), labeled as ACC, SN, and SP, respectively, in the figure. Prevalence and accuracy were 24% and 86%, respectively. Sensitivity and specificity were 83.3% (95% CI: 51.5–97.9%) and 86.8% (95% CI: 71.9–95.6%), respectively. Positive and negative likelihood ratios were 6.33 (95% CI: 2.7–14.9) and 0.19 (95% CI: 0.05–0.7), respectively. In Table 1, the diagnostic performance of the present CT-iFR method is compared with that of CT-FFR reported in the literature. All data are from studies of per-vessel basis. One of the CT-FFR studies in Table 1 was written by our group (Chung *et al*.^22^); the others used the method of HeartFlow, Inc.^16–18,24^ (Redwood City, CA, USA). The number of vessels in the present CT-iFR study was 50, which was somewhat less than those of other studies, with the exception of two^23,24^. Among the clinical validation studies of CT-FFR shown in Table 1, the NXT study showed better clinical performance than the other studies. The clinical performance of our current CT-iFR study approached that of the NXT study in terms of sensitivity, specificity, accuracy, and AUC. Moreover, in terms of Pearson’s correlation coefficient, the present CT-iFR study (0.85) showed more accurate results than the NXT study (0.82).

**Table 1:** Diagnostic performance comparison of the present CT-iFR method with CT-FFR performance from published works^16–18,22–24^.

Study name	Reference	No. of vessels	Sensitivity	Specificity	Accuracy	Area under curve (AUC)	Pearson’s correlation coefficient
DISCOVER-FLOW (CT-FFR)	Koo *et al*.^16^	159	88%	82%	84%	0.90	0.72
DeFACTO (CT-FFR)	Min *et al*.^17^	407	80%	63%	69%	0.81	0.63
NXT (CT-FFR)	Norgaard *et al*.^18^	484	84%	87%	86%	0.93	0.82
CT-FFR study	Kim *et al*.^24^	48	85%	57%	77%	—	0.60
NOVEL-FLOW	Chung *et al*.^22^	218	86%	86%	85%	0.93	0.76
Previous other CT-iFR study	Ma *et al*.^23^	47	70.6	83.3	78.7	0.87	0.75
Present CT-iFR study		50	83.3	86.8	86%	0.93	0.85

## Discussion

FFR is a gold standard for PCI strategy and is measured over an entire cardiac cycle under a hyperemic state induced by adenosine infusion. Recently, another physiological index, iFR, was proposed for clinical PCI strategy. Unlike FFR, iFR is measured during a wave-free period of the cardiac cycle under non-hyperemic resting conditions^4^. Recent clinical investigations published in the *New England Journal of Medicine* reported that iFR is comparable to FFR, in terms of diagnostic performance in PCI strategy^12,13^. Now, a variety of clinical studies to delineate the diagnostic characteristics of the iFR index are currently underway. Along with invasive measurement of iFR, a noninvasive computational approach, CT-iFR, has been proposed. CT-iFR is a very powerful diagnostic technique for specific patients, for example, those without hemodynamic response to intravenous adenosine, as well as for patients with ventricular hypertrophy and other conditions. Notably, the resting state is significantly associated with clinical outcome. However, there has been no clinical validation study of CT-iFR. Considering that CT-FFR was used in the PCI strategy after several clinical validation studies^16–18^, evaluation of the diagnostic performance of CT-iFR is a prerequisite for its clinical application.

**Figure 5:**
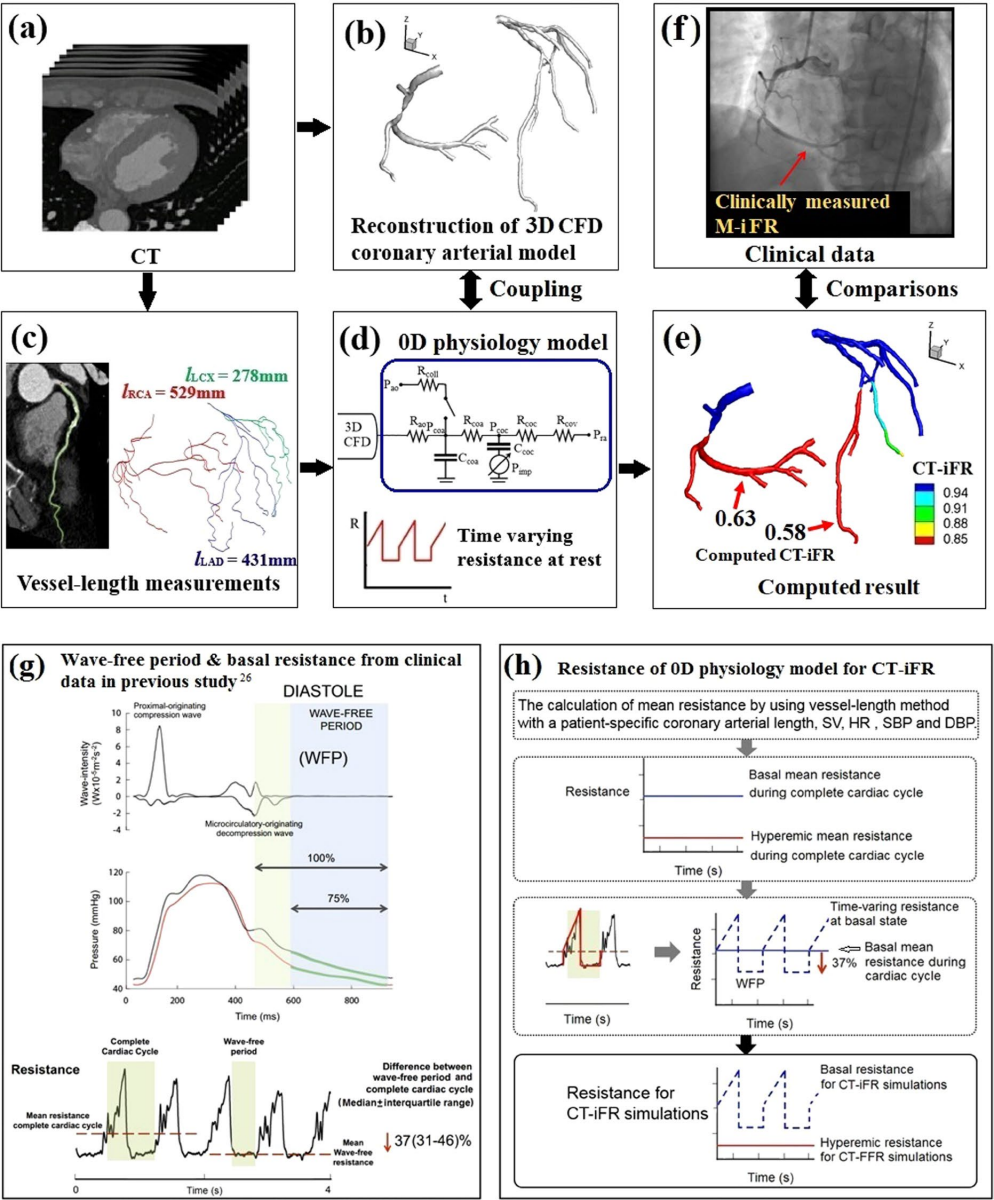
Procedure of computer simulation to compute CT-iFR. (**a**) Patient-specific coronary computed tomography (CT) angiogram acquisition. (**b**) Reconstruction of three-dimensional (3D) computational fluid dynamics (CFD) coronary model from CT images. (**c**) Coronary vessel length measurement from CT, where *l* is the vessel length of ‘LCX,’ ‘LAD,’ and ‘RCA, referring to the left circumflex artery, left anterior descending artery, and right coronary artery, respectively. (**d**) Lumped parameter model (LPM) establishment using a vessel-length based method. LPM consists of pressure ‘P’, resistance ‘R’, and capacitance ‘C’ elements. The subscripts for ‘P,’ ‘R,’ and ‘C’ are ‘ao,’ ‘coll,’ ‘coa,’ ‘coc,’ ‘cov,’ and ‘ra,’ indicating aorta, collateral circulation, coronary arterial compartment, coronary capillary compartment, coronary venous compartment, and right atrium, respectively. Basal time-varying resistance was used for CT-iFR simulations. (**e**) Computed CT-iFR results obtained by coupling between the 3D CFD model and the LPM. (**f**) Clinical validation by comparing the computed iFR results with the clinically measured iFR data at the identical location. (**g**) Wave-free period, pressure waveform, and time-varying resistance pattern from clinical data of a previous paper^25^ (these figures are reproduced from Figs. 1 and 7 of ref. ^25^). (**h**) Non-hyperemic time-varying resistance establishment for the LPM of CT-iFR by approximating trapezoidal patterns.

There are two main contributions of the present study. First, we propose a vessel-length-based CT-iFR method. This method is similar to the CT-FFR method^20,21^ applied in our earlier study. In the CT-iFR method of the current study, a patient-specific 3D CFD model is combined with the vessel-length-based LPM of coronary circulation. Under hyperemic conditions for the CT-FFR calculation, the microvascular resistance of the LPM is a constant value over the entire cardiac cycle, as shown in Fig. 5(h). However, in the CT-iFR method, the resistance is a time-varying value under non-hyperemic resting conditions, as shown in Fig. 5(h). As far as we know, there has been no microvascular resistance model that takes into account such time-varying characteristics. To resolve this problem, we propose a simplified time-varying trapezoidal resistance method, mimicking existing clinical observations^25^ [Fig. 5(g)]; this method was tested to determine its validity via clinical outcome verification.

Second, we found that the clinical diagnostic performance of CT-iFR was comparable to that of CT-FFR. Here, CT-iFR simulations were performed for 50 vessels in 32 patients for comparisons with clinically measured M-iFR data (Table 1). Of the 32 patients, 11 patients tested positive (M-iFR ≤ 0.89). The number of positive vessel samples was 12 among the 50 vessels in Fig. 1(a). CT-iFR simulations showed a diagnostic performance with an AUC of 0.93 on a per-vessel basis (Fig. 3), indicating good diagnostic performance in CT-iFR computations. Among the validation studies of computed FFR (CT-FFR) in Table 1, the NXT study^18^ showed better clinical performance over the criteria specified, with the exception of sensitivity, compared with the other studies. The clinical performance of the present CT-iFR method almost equalled that of the NXT study in terms of sensitivity, specificity, and accuracy. Moreover, Pearson’s correlation coefficient of the present CT-iFR study was 0.85, indicating more accurate results than the coefficient determined in the NXT study of 0.82. However, in terms of sensitivity, the present CT-iFR method was inferior to CT-FFR methods, although the sensitivity of the DeFACTO study was lower.

Our study had several other limitations. First, the number of patients with measured iFRs was relatively small. As shown in Table 1, the present CT-iFR study had a lower vessel number than most of the CT-FFR studies, except for the studies of Ma *et al*.^23^ and Kim *et al*.^24^. The computed iFR obtained by the current method showed better diagnostic performance than the previously computed iFR by Ma *et al*.; however, it would be difficult to compare the diagnostic performance with the previous one^23^, as both studies were performed in relatively small study populations and for selected population samples. Therefore, our future work will include a larger number of blood vessels to validate the CT-iFR approach. Second, the patient-specific time-varying resistance of the microvascular model is not perfect. An observed microvascular resistance pattern in 51 patients in the previous study^25^ was used for the time-varying resistance pattern of this study, regardless of the patients’ specific condition. However, variations in patients’ conditions may need to be considered in certain cases (e.g., multi-vessel disease, diffuse disease, advanced micro-vessel circulation disease, etc.); thus, this is a limitation of this study. For multi-vessel diseases, increasing the population of this patient group is necessary. However, the numbers for this patient group do not tend to reach the required enrollment quickly; thus, the study would have to be conducted over a longer term. For diffuse diseases, the mean stenosis length and mean stenosis degree in diffuse stenosis are important factors in understanding the effects on the microcirculatory system; these two factors are also necessary for clinical studies. Influences on the microcirculatory system can be studied by grouping patients according to the stenosis length and the stenosis degree. However, long-term investigations should be planned for this. Third, identifying the correct stenosis severity and correct lesion length can be difficult in calcified vessels. This is because calcification produces image noise, and the calcified area can be misinterpreted as arterial lumen. In this study, two calcified vessels were included. The area of lumen was overestimated; thus, the CT-iFR reading was overestimated compared with that using M-iFR. Calcification may degrade the performance of noninvasive iFR, particularly for patients of an advanced age. In this study, a small number of calcified vessels was included. Increasing the number of calcified vessels may increase the misdiagnosis of stenosis severity; however, the limited number of calcified vessels was also a limitation of the current study. Fourth, we did not use a turbulent model (i.e., a k-ε model); nevertheless, turbulent effects may not be negligible in vessels with severe stenosis. The absence of a turbulence model in this study may have led to overestimated CT-iFR values, compared with clinical iFR measurements. Therefore, the iFR value might be lowered further and might show better diagnostic performance if the turbulence effect were taken into account. Lastly, there are difficulties in clarifying the causes of error in individual CT-iFR calculations. Errors can arise from many sources in CT-iFR. For example, segmentation errors derived from images due to image resolution or a calcified vessel, etc.; errors derived from the vessel-length method; errors derived from neglecting advanced microcirculatory disease; and 4) errors derived from neglecting the turbulence effect in severe stenosis cases. Thus, given the potential error from all of these sources, it is not simple to estimate the error separately for individual vessels. We attempted to determine the error on a case by case basis.

We do not consider that these limitations significantly influenced our major findings. To our knowledge, this is the first report in which CT-iFR values were compared with clinically measured iFR data (M-iFR) for clinical validation. The diagnostic performance evaluation in this study shows that CT-iFR can be used as a pre-operative aid to establish PCI strategy as a noninvasive alternative to M-iFR.

## Methods

### Study population and clinical measurements

In total, 51 vessels of 33 patients underwent coronary CT angiography and invasive iFR; however, 1 patient was excluded due to the poor diagnostic quality of a blurred CT image [Fig. 1(a)]. Therefore, we used a sample set of 50 vessels from 32 patients who underwent coronary CT angiography examinations and iFR measurements at Ulsan University Hospital (Ulsan City, Republic of Korea) (Fig. 1). The inclusion criteria were: patients who were at least 18 years old and suspected of having coronary artery disease, and who had > 30% stenosis by visual estimation in major epicardial coronary arteries and underwent successful iFR measurement. Coronary X-ray angiography, coronary CT angiography, and iFR examinations followed current guidelines^1,2,25,26^. For the coronary CT, we used a dual-source CT scanner platform (Definition CT, Siemens, Forchheim, Germany; Aquilion ONE, Toshiba, Otawara, Japan). The scanning parameters were: tube voltage, 100 kV; tube current, 320 mA; CT slice thickness, 0.6 mm; and number of pixels, 512 × 512. Scans were performed with prospective electrocardiogram triggering. The numbers of coronary CT slices obtained ranged from 300 to 600 slices. For CT acquisition, sublingual nitroglycerin was given, and β-blockers were administered to patients with a rapid heart rate, according to the Society of Cardiovascular Computed Tomography (SCCT) guidelines^26^. Contrast agent was injected, followed by a saline flush. CT was scanned for all coronary arteries, including the left ventricle and proximal ascending aorta. iFR measurements were also made according to current guidelines^26^. Cardiac catheterization was performed via a femoral approach. At the start of the procedure, unfractionated heparin (5000 IU) was given intravenously. A 0.014-inch pressure-sensor-tipped wire (PrimeWire Prestige; Volcano Corporation, San Diego, CA, USA) was positioned at the tip of a guiding catheter. After intracoronary administration of nitrate and pressure equalization at the tip of the catheter, the wire was advanced into the target vessel as distally as reasonably possible for pressure recordings. Physiological data for iFR were calculated automatically online using a commercially available system (S5 Imaging System; Volcano Corporation). iFR, the ratio of the mean distal coronary pressure to the mean proximal pressure during the wave-free period, was obtained using a catheter guide-wire (see Fig. 1 of refs. 4, 6, and  25)^4,6,25^. An iFR value ≤ 0.89 is considered the cut-off value to indicate significant myocardial ischemia^27^.

### Numerical models and methodology

The main procedures of this study to compute CT-iFR are shown in Fig. 5. This method was originally proposed in our previous paper^19^ in 2014 and has been validated clinically in our subsequent studies^20,21^. More details were explained in the previous studies^19–22^. A non-invasive computer simulation method to evaluate the iFR value is based on patients’ CT images and the physiological data. The method uses a CFD approach for the hemodynamics of the coronary arteries coupled with the LPM of the coronary circulation system, as in our previous papers^20–22^. To calculate the LPM parameters, such as resistances and capacitances, we employed the novel vessel-length-based method^21^. We measured the vascular centerline length from CT images. Then, we first obtained each summed length of the LAD, LCX, and RCA. A detailed explanation is given in our previous papers^20–22^. In the equations, resistance, denoted as ‘R’, is inversely proportional to vessel length, *l*, indicating that a longer vessel length induces less resistance and thus more blood flow. Here,α is set to 3.45, reducing the effect of the RCA vessel length feeding the right ventricle (RV) muscle on RCA flow. ‘k’ is a proportionality constant derived from the pressure-flow rate relation, as explained in our previous paper^21^:

1 $${R}_{LAD}=\frac{k}{{l}_{LAD}},$$

2 $${R}_{LCX}=\frac{k}{{l}_{LCX}},$$

3 $${R}_{RCA}=\frac{\alpha k}{{({l}_{RCA})}_{RV}}+\frac{k}{{({l}_{RCA})}_{LV}},$$

4 $${R}_{LAD}=\frac{k}{{l}_{LAD}},\,{R}_{LCX}=\frac{k}{{l}_{LCX}},\,{R}_{RCA}=\frac{\alpha k}{{({l}_{RCA})}_{RV}}+\frac{k}{{({l}_{RCA})}_{LV}},$$

5 $$\begin{array}{rcl}{Q}_{total} & = & {CO}\ast 0.04\,\,(CO:\,{\rm{cardiac}}\,{\rm{output}}),\\  & = & {Q}_{LAD}+{Q}_{LCX}+{Q}_{RCA},\end{array}$$

6 $${Q}_{total}=\frac{({P}_{aorta}-{P}_{vein})}{{R}_{LAD}}+\frac{({P}_{aorta}-{P}_{vein})}{{R}_{LCX}}+\frac{({P}_{aorta}-{P}_{vein})}{{R}_{RCA}}.$$

In Eq. (6), *P*_*aorta*_ is calculated from the measured blood pressure of the patient, and *P*_*vein*_ is assumed to be 2 mmHg. *Q*_*total*_ is calculated from the cardiac output (*CO*), which is based on the measured heart rate and stroke volume. If we substitute *R*_*LAD*_, *R*_*LCX*_, and *R*_*RCA*_ of Eq. (6) with the values obtained from Eq. (4), then we can obtain the value of ‘*k*’, a patient-specific proportionality constant.

**Figure 6:**
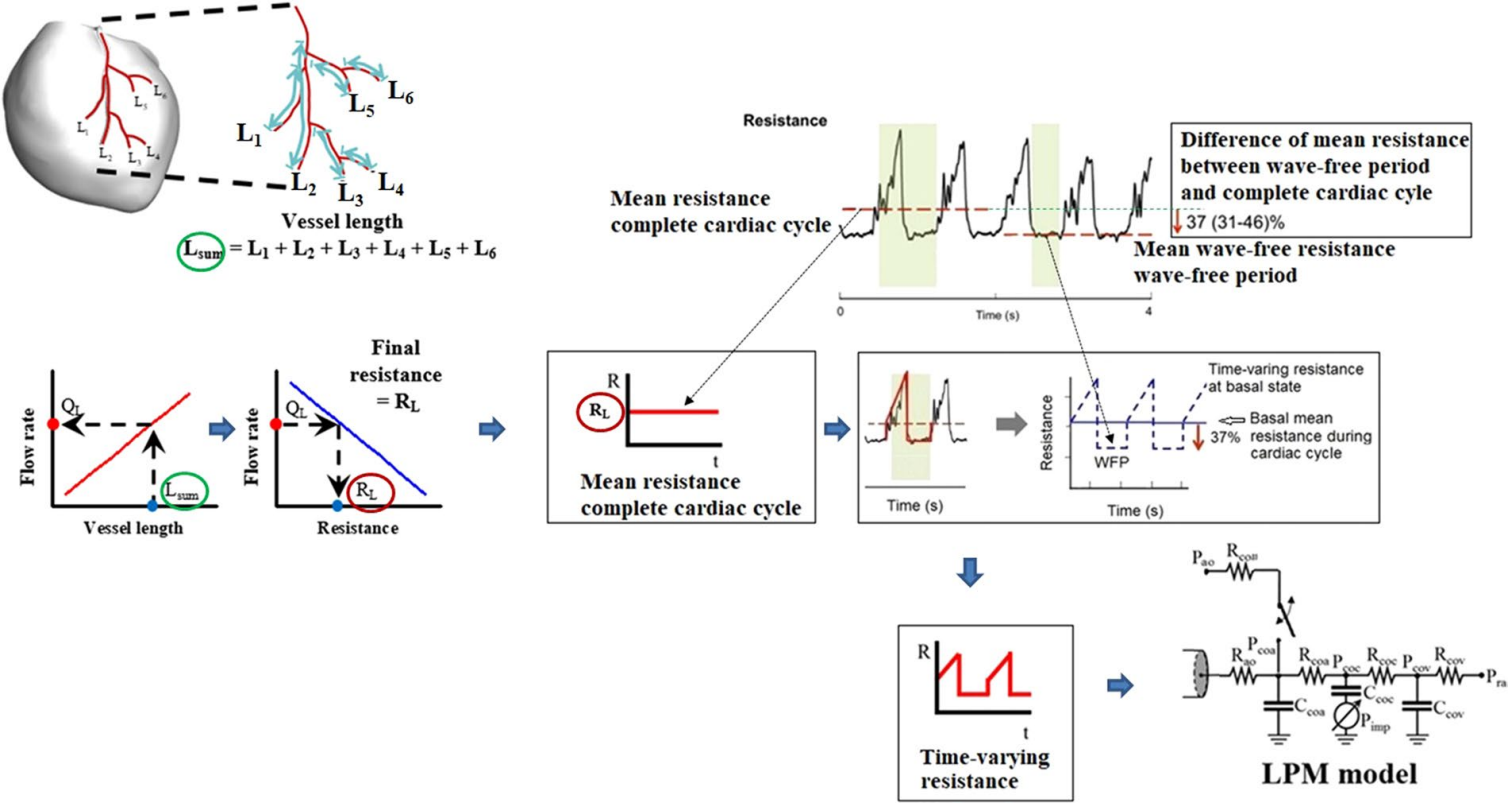
Schematic diagram of the vessel length method to estimate microvascular resistance of a coronary vessel at rest. The inset of the resistance plot (right upside panel) was copied from ref. ^25^.

A schematic diagram of the present vessel-length method to estimate LPM resistance at rest is shown in Fig. 6. The LPM for the coronary circulation is used to represent the coronary vasculature from the small-size coronary arteries to the right atrium (Figs. 5 and 6). In the LPM, the coronary circulation involves three compartments: the coronary arteries (subscript coa), capillaries (subscript coc), and veins (subscript cov) [Fig. 5(d)]. The effect of myocardial squeezing by ventricular muscle contraction is approximated by the intra-myocardial pressure (subscript imp), and collateral flow (subscript coll) is considered using the switching system. The model is formulated in terms of an electric analog model consisting of elements such as resistors, capacitors, and a diode.

**Figure 7:**
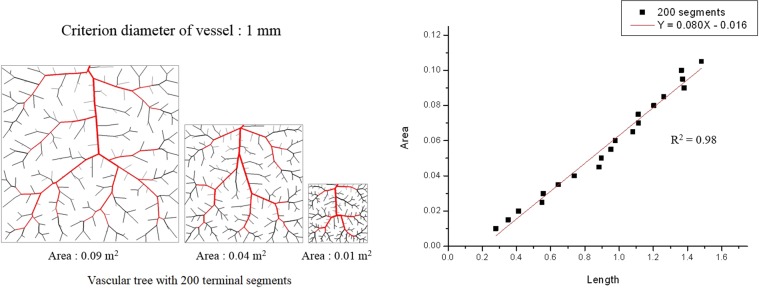
Optimized vascular fractal tree modeling in various areas (e.g., 0.09 m^2^, 0.04 m^2^, and 0.01 m^2^) (left panel), and the linear relationship between the area (volume) and the total length of the blood vessel (right panel).

To reconstruct the geometry of the 3D CFD model, CT image slices were segmented using semi-automated commercial software (Aquarius, ver. 4.4.11; TeraRecon, Inc., Foster City, CA, USA). We reconstructed the vessel based on the identification of the vessels in CT images by a lumen cross-sectional area of 1.5 mm^2^. We applied this value to all vessels, given that there is a linear relationship between the area (volume) and the total length of a blood vessel (the right graph of Fig. 7), when creating a network of small arterial blood vessels that supply blood flow to a certain area (or volume) (left panel of Fig. 7). The figure is based on an optimized vascular fractal tree modeling technique^28^ and the length of the artery above a certain diameter, as shown in red (the left panel in Fig. 7). From this correlation we can observe important physiological principles. In a particular artery branch, measuring the total length of blood vessels belonging to that branch can infer the total area (or volume) of tissue covered by the branch. However, its volume is directly proportional to the blood flow that the branch feeds. Thus, this information can be used to determine the endovascular resistance of that branch. For mesh generation of the reconstructed 3D model, a spatial resolution (mesh size for the 3D model) was approximated as 0.0001 m, and the number of elements was estimated to be in the range of 3 × 10^6^ to 2 × 10^7^. The temporal resolution (time resolution of the time steps) was 0.002 s.

Numerical simulations of blood flow behavior in the 3D CFD model were performed using a Fortran-based in-house code, an incompressible Navier–Stokes solver, based on a segregated finite-element method^19^. The inlet boundary conditions for the simulation were estimated from the aortic pressure from clinically measured blood pressure and the heart rate from a patient’s brachial artery^20,21^. Briefly, local hemodynamics in a 3D CFD model were coupled with global hemodynamics in the vessel-length-based LPM by an iterative method to update the pressure and flow rate^19^. In the iterative method, in each time step the computed flow rate in the outlet of the 3D CFD model was transferred to the LPM. We calculated the pressure and the flow rates in the LPM by solving the ordinary differential equations. The computed pressure in the LPM was used for the outlet condition of the 3D CFD model.

**Figure 8:**
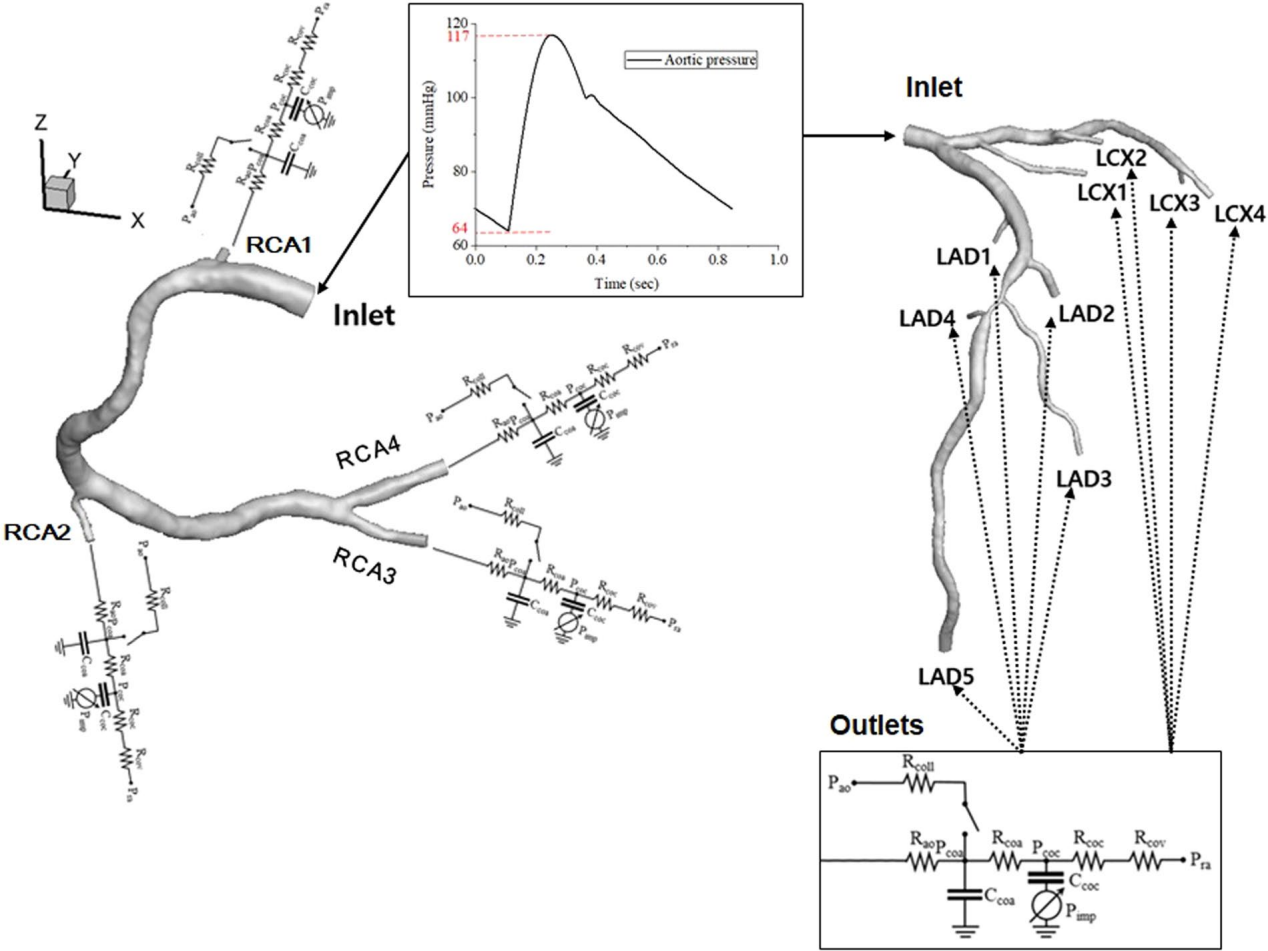
Representative 3D patient-specific CFD model, including inlet and outlet conditions (parameters in Tables 2–4).

**Figure 9:**
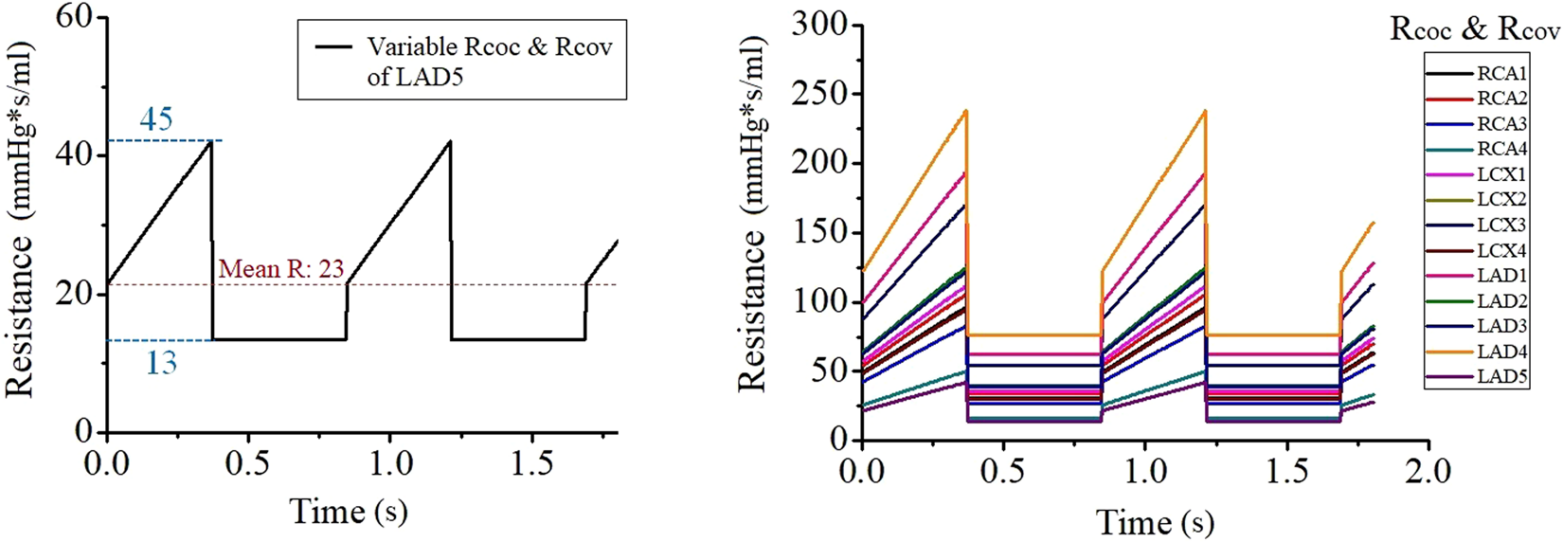
Time-varying Rcoc and Rcov [resistances for capillaries (subscript coc) and veins (subscript cov)] patterns in the model of Fig. 8. Time-variable Rcoc and Rcov patterns of LAD 5 (left panel). Time variable Rcoc and Rcov patterns of each branch (right panel) (Unit: R, mmHg∙s/mL; C, mL/mmHg).

More details of these patient-specific parameters used in the CFD are presented using a representative case (Figs. 8 and 9, and Tables 2–4) that included the ratio of the length of the RCA, LAD, LCX, blood pressure, stroke volume, heart rate, hematocrit ratio, etc. Figure 8 shows a representative case of a 3D patient-specific CFD model coupling the inlet and outlet conditions. Table 2 shows patient-specific physiological parameters and Table 3 shows the patient-specific length ratio of RCA, LAD, and LCX. Table 4 presents the LPM of Fig. 8 that includes the mean values of vessel-specific resistances (R) and capacitances (C) (units: R, mmHg∙s/mL, C, mL/mmHg). Figure 9 shows the time variable Rcoc and Rcov [resistances for capillaries (subscript coc) and vein (subscript cov)] patterns in the model of Fig. 8. The left panel shows the time-variable Rcoc and Rcov patterns of LAD 5, and the right panel shows the time-variable Rcoc and Rcov patterns of each branch. The patient-specific proportionality constant, k, was 21673 for the model shown in Fig. 8. The spatial resolution (mesh size for the 3D model) was approximately 0.0002 m, and the number of elements was approximately 8.6 × 10^5^. The temporal resolution (time resolution of the time step) was 0.002 s. Figure 4(b) displays the CT-iFR results in the coronary model of Fig. 8. A more detailed explanation of the coupling method between the 3D CFD model and the LPM can be found in our previous papers^19–21^.

**Table 2:** Patient-specific physiological parameters in the coronary model of Fig. 8.

SBP(mmHg)	DBP(mmHg)	HR	Stroke volume(ml)	Hematocrit(%)
117	64	71	67	40.1

HR: heart rate.

**Table 3:** Patient-specific length ratio of RCA, LAD, and LCX in the coronary model of Fig. 8.

Artery	RCA	LAD	LCX
Length (mm)	353.5	323.9	228.5

RCA: right coronary artery; LAD: left anterior descending artery; LCX: left circumflex artery.

**Table 4:** Lumped parameter model in the coronary model of Fig. 8 (the mean values of vessel-specific resistances (R) and capacitances (C) (units: R, mmHg∙s/mL; C, mL/mmHg).

	Mean of R_ao_	Mean of R_coa_	Mean of R_coc_ & R_cov_	Mean of C_coa_	Mean of C_coc_
RCA1	98.074	159.370	49.037	0.000312	0.005932
RCA2	107.464	174.629	53.732	0.000285	0.005414
RCA3	84.283	136.960	42.141	0.000363	0.006903
RCA4	51.009	82.890	25.505	0.000600	0.011406
LCX1	113.691	184.747	56.845	0.000269	0.005117
LCX2	125.138	203.349	62.569	0.000245	0.004649
LCX3	173.631	282.150	86.815	0.000176	0.003351
LCX4	96.146	156.237	48.073	0.000318	0.006051
LAD1	197.201	320.452	98.601	0.000155	0.002950
LAD2	127.300	206.863	63.650	0.000241	0.004570
LAD3	124.130	201.712	62.065	0.000247	0.004687
LAD4	242.554	394.150	121.277	0.000126	0.002399
LAD5	46.283	75.210	23.141	0.000662	0.012570

### Statistical analyses

Comparisons were made between clinical data (M-iFR) and computed values (CT-iFR) for validation. All data are expressed as the median and the interquartile range (Q1–Q3). We analyzed data on a per-vessel basis using commercial software (OriginPro 8, Northampton, MA, USA, and MedCalc, Mariakerke, Belgium). In a per-vessel basis analysis, 50 measured M-iFR data and computed CT-iFR values were included. The extent of the agreement between computed and clinical indices was evaluated by drawing Bland–Altman plots and scatter plots. We plotted ROC curves for CT-iFR, assuming an iFR cut-off value of 0.89 as the reference standard. The performance of CT-iFR is shown in terms of AUC, sensitivity and specificity with 95% confidence intervals.
